# Radiosynthesis of [^**18**^F]Trifluoroalkyl Groups: Scope and Limitations

**DOI:** 10.1155/2014/380124

**Published:** 2014-07-10

**Authors:** V. T. Lien, P. J. Riss

**Affiliations:** ^1^Kjemisk Institutt, Universitetet I Oslo, Sem Sælands Vei 26, 0376 Oslo, Norway; ^2^Norsk Medisinsk Syklotronsenter AS, Postboks 4950 Nydalen, 0424 Oslo, Norway

## Abstract

The present paper is concerned with radiochemical methodology to furnish the trifluoromethyl motif labelled with ^18^F. Literature spanning the last four decades is comprehensively reviewed and radiochemical yields and specific activities are discussed.

## 1. Introduction

Substantial interest has been given lately to the trifluoromethyl group in the context of radiotracer development for positron emission tomography (PET). PET imaging of radiotracer distribution in living systems provides noninvasive insights into biochemical transactions in vivo. PET relies on molecular probes labelled with positron emitting radionuclides that participate in the process of interest. Coincident detection of 511 keV photons originating from positron-electron annihilation allows for spatial localisation and quantification of the decaying radionuclide in tissue with some accuracy [1–4]. Application of PET in biomedical research, drug development, and clinical imaging creates an immanent need for radiotracers for a variety of biological targets.

The neutron-deficient fluorine isotope ^18^F is the most frequently employed PET nuclide [5]. This is due to an expedient half-life of 109.7 min, which facilitates commercial distribution of ^18^F-radiotracers and permits convenient handling of the tracer in multistep reactions and imaging studies [1–3]. Almost exclusive decay via the *β* ^+^ decay branch (97%), paired with a very low positron energy (638 keV), effectively limits the linear range of the emitted positron in water and makes up for the highest image resolution compared to most other PET nuclides. ^18^F readily forms stable bonds to carbon atoms, which promotes the straightforward introduction of F atoms into most organic molecules. Using the ^18^O(p,n)^18^F nuclear reaction, batch production of 370 GBq [^18^F]fluoride ion (10^3^ human doses) is routinely achievable by irradiation of H_2_^18^O liquid targets. High specific radioactivity (*A*_*s*_; [*A*_*s*_] = Bq/mol; >150 MBq/nmol) is a realistic expectation for radiotracers prepared from [^18^F]fluoride ion. Likewise, high specific activity (>100 MBq/nmol) is inevitable to maintain genuine tracer conditions for PET-imaging of saturable biological systems [1, 4–7], particularly in small animals but even so in human subjects [8]. As such, high specific activity is a cornerstone of the tracer principle, first introduced by George de Hevesy [1, 6, 9]. In theory, PET may provide the means for noninvasive observation of chemical processes in tissues, exemplified here for receptor binding, as long as the injected amount of the radiotracer does not lead to significant receptor occupation (RO). Receptor occupation is closely linked to pharmacodynamic efficacy and a pharmacological effect can be omitted in cases when the RO is negligible. Figures for insignificant RO have been specified to <1%–5%; nevertheless, it has to be kept in mind that RO is a specific characteristic of a receptor-ligand system and thus may vary from target to target [6, 7, 9]. Hence, the specific activity of each individual radiotracer has to meet the requirements for noninvasive PET imaging. For these reasons, the* A*_*s*_ is the most important quality measure for a labelling reaction rather than chemical or radiochemical yield.

Evidently, an abundant motif such as the trifluoromethyl group and its presence in a large number of agrochemicals and biologically active drug molecules is of tremendous interest for the PET community. Consequently, earliest attempts to access this group by nucleophilic and electrophilic radiofluorination protocols date back into the very beginnings of the field of PET chemistry (see Table 1 for an overview).

Classical organic chemistry has seen a surge in the development of novel trifluoromethylation strategies and protocols [29–35], owed to the high relevance of the trifluoromethyl motif [36, 37]. However, translation of these most useful and often robust and versatile protocols into radiochemistry is not without difficulties. Indeed, straightforward translation of known organic reactions under stoichiometric conditions into no-carrier-added nucleophilic radiosynthesis often precipitates in adverse findings. Polyfluorinated, organic moieties complicate nucleophilic radiofluorination using ^18^F-fluoride ion. Under the common conditions used, there is an inherent vulnerability for isotopic dilution of the labelled product with its nonradioactive analogue. Inherently unproblematic exchange processes between carbon-bound ^19^F and ^19^F anions in the reaction mixture, which do not confound the final quality of a nonradioactive product, can devastate the specific activity of PET radiotracers [38].

## 2. Nucleophilic Radiosynthesis

Nucleophilic radiofluorination remained rarely a successful protocol prior to the advent of supramolecular chemistry and the development of potassium selective cryptands. The latter, when combined with mild organic potassium bases and aqueous solutions of ^18^F^−^ proved to be key to improve the inherently low solubility and reactivity of fluoride ion in dipolar aprotic solvents [39, 40].

**Figure 1:**
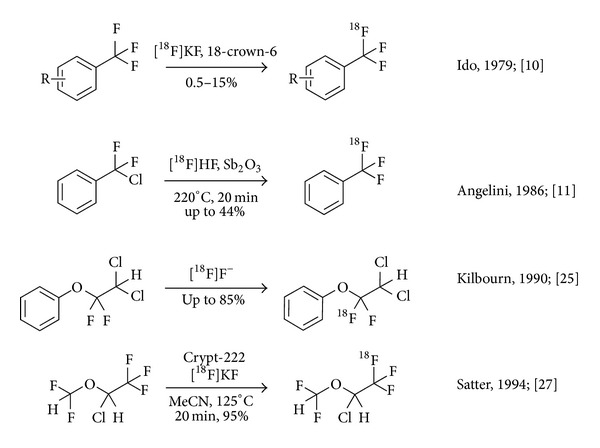
Nucleophilic radiosynthesis of ^18^F-labelled trifluoroalkyl groups using isotopic exchange and antimony mediated ^18^F-for-Cl substitution.

Earliest attempts of developing suitable methodology for the synthesis of the [^18^F]CF_3_ group involved transient incorporation of an ^18^F label into trifluoromethylated scaffolds via ^18^F-^19^F isotopic exchange at high temperature as devised by Ido et al. [10]. Shortly thereafter, Lewis acid catalysed dechlorofluorination of chlorodifluoromethyl groups via a straightforward protocol using H^18^F and Sb_2_O_3_ was utilised in the synthesis of ^18^F-labelled trifluoromethyl arenes [11, 12]. Both of these procedures afforded the desired products in only moderate yields and low specific activity. Nevertheless, isotopic exchange protocols were soon found to be reliable protocols to achieve radiofluorination of di and tri-fluorinated carbon centres, somewhat tolerant to the presence of water [13], albeit with strict limitation for the achievable specific activity, governed by the fact that only a fraction of the obtained carrier-added product will actually contain the radiolabel (see Figure 1).

However, low specific activity may not be an issue in PET studies targeting physical or metabolic processes in vivo, for example, in the case of the mechanisms of action of fast-acting aerosols for anaesthesia. Similarly, radiolabelling of chlorofluorocarbon (CFC) replacement agents such as 1,1,1,2-tetrafluoroethane (HFA 134a) for radiotracer studies does not require particularly high specific radioactivities. This fact was exploited by Satter et al. and Aigbirhio et al. who labelled the CFC replacement agent HFA 134a using an isotopic exchange reaction on the nonradioactive analogue [14, 16, 41, 42]. Satter et al. studied the isotopic exchange between ^18^F and ^19^F as a means for the synthesis of ten inhalation anaesthetics, including isoflurane and halothane, all of which possess a trifluoromethyl group. Labelling was achieved by heating the corresponding inhalants with potassium-4,7,13,16,21,24-hexaoxa-1,10-diazabicyclo[8.8.8]hexacosane (crypt-222) cryptate ^18^F complex in dimethyl sulfoxide or acetonitrile in high yields.

Various reported products were subsequently studied in man, dog, and one compound; namely, [^18^F]HFA-134a was studied in rat using PET (Figure 1) [42].

**Figure 2:**
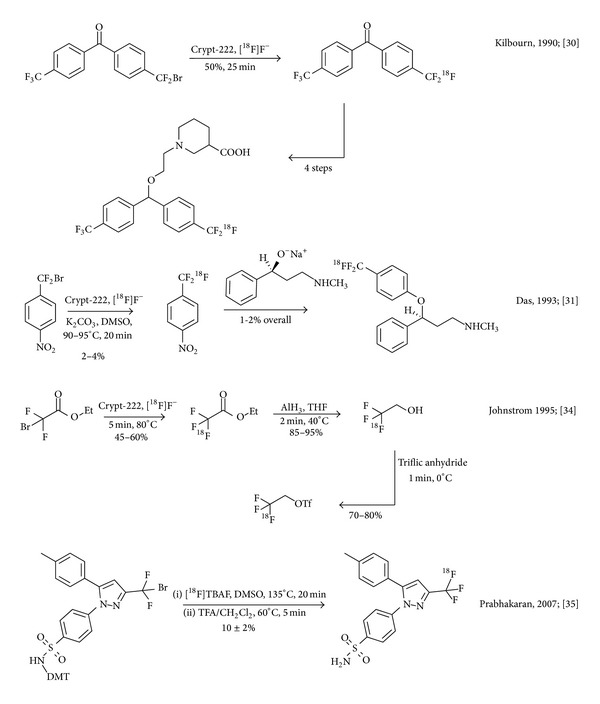
^18^F-for-Br nucleophilic substitution protocols yielding the [^18^F]CF_3_ motif.

Kilbourn et al. then resorted to a classical ^18^F-for-Br nucleophilic substitution procedure to obtain [^18^F]trifluoromethyl arenes under no-carrier-added conditions in an elaborate multistep protocol. These researchers successfully established direct nucleophilic radiofluorination of a suitable difluorobenzylic bromide, which was subsequently employed as a building block in the radiosynthesis of an ^18^F-labelled GABA transporter ligand. The [^18^F]trifluoromethylated product was obtained in 17–28% radiochemical yield; decay corrected to the end of bombardment after a synthesis time of 150 minutes over 5 steps. In the original publication, the authors describe that the specific activity in this procedure is confounded by the presence of inseparable labelling precursor which is surmised to act as a biologically active pseudocarrier (Figure 2) [17].

Further insights into the nucleophilic radiofluorination of bromodifluoromethyl-precursors were obtained by Mukherjee and Das in 1993, when the team studied radiolabelling of the selective serotonin uptake inhibitor (S)-*N*-methyl-*γ*-[4-(trifluoromethyl)phenoxy]benzenepropanamine (fluoxetine) to obtain [^18^F]fluoxetine as a potential radiotracer for serotonin transporter binding sites. Radiolabeling of *α*-bromo-*α*,*α*-difluoro-4-nitrotoluene with no-carrier-added [^18^F]fluoride ion was described in low 2–4% yields and low specific activity of 2.59 MBq/nmol. A strong effect of the reaction temperature on the radiochemical yield and specific activity of the product were studied and a negative correlation between temperature and specific activity was observed. At higher temperatures, products were found to contain a larger amount of nonradioactive carrier, which had been formed in the reaction. Overall decay-corrected yields over 2-steps spanning a total radiosynthesis time of 150–180 min were 1-2%. The specific activity of the product was 1.48 MBq/nmol (Figure 2) [15, 43].

In parallel, Hammadi and Crouzel investigated radiosynthesis of [^18^F]fluoxetine with [^18^F]fluoride ion for PET imaging studies [44]. Radiosynthesis was achieved in a decay-corrected radiochemical yield of 9-10% and a specific radioactivity of 3.70–5.55 MBq/nmol within 150 min from the end of bombardment. A competing isotopic exchange reaction was demonstrated, which the authors suspected to reduce the specific activity of the final ^18^F-labelled product.

Contrary to isotopic exchange reactions, wherein the factors limiting specific activity are evidently linked to the deliberate addition of nonradioactive carrier, the* A*_*s*_ limiting factors in the studied nucleophilic substitution reaction are less apparent. Given that a temperature dependency of specific activity was observed by Das and Mukherjee, two principle mechanisms come to mind: (a) dilution through isotopic exchange with the precursor, which would effectively sacrifice ^18^F to a labelled precursor molecule and yield a ^19^F fluoride ion that could in itself react with the precursor and (b) degradation of a fluorinated component in the reaction mixture to afford a nonradioactive degradation product and free nonradioactive fluoride ion. However, we refrain from hypothesizing about nonvalidated theories within this paper.

Nevertheless, Johnström and Stone-Elander encountered what appeared to be an example of case (b) during attempts to synthesize the alkylating agent 2,2,2-[^18^F]trifluoroethyl triflate via the nucleophilic reaction of [^18^F]F^−^ with ethyl bromodifluoroacetate [18]. These researchers were alerted by the observation of unlabeled ethyl trifluoroacetate produced from ethyl 2-bromo-2,2-difluoro-acetate which reduced the specific activity of the product to only about 0.04 MBq/nmol [38]. Ethyl [^18^F]trifluoroacetate was synthesized from [^18^F]F^−^ and ethyl bromodifluoroacetate in DMSO (45–60%, 5 min, 80°C) followed by distillation in a stream of nitrogen. Despite the use of no-carrier-added [^18^F]F^−^, the specific activity of the final product was found to be 0.037 MBq/nmol.

Attempts to mitigate the amount of ^19^F released from the substrate during the reaction were only partially successful. Even under optimised conditions, the specific activity remained below 1 MBq/nmol, which is roughly two to three orders of magnitude below routinely achievable figures associated with direct nucleophilic substitution reactions with [^18^F]fluoride ion on aliphatic or aromatic carbon centres (Figure 2).

Bromodifluoromethyl precursors were reconsidered in a more recent radiosynthesis of the selective COX-2 inhibitor 4-[5-(4-methylphenyl)-3-([^18^F]trifluoromethyl)-1*H*-pyrazol-1-yl]benzenesulfonamide ([^18^F]celecoxib). [^18^F]celecoxib was obtained using [^18^F]TBAF in DMSO at 135°C in 10 ± 2% yield with >99% radiochemical purity and a specific activity of about 4.4 ± 1.5 MBq/nmol (EOB). Although the CNS distribution of [^18^F]celecoxib mirrored COX-2 expression in the primate brain, the radiotracer was found to be susceptible to defluorination in vivo in rodent and baboon PET studies [20].

**Figure 3:**
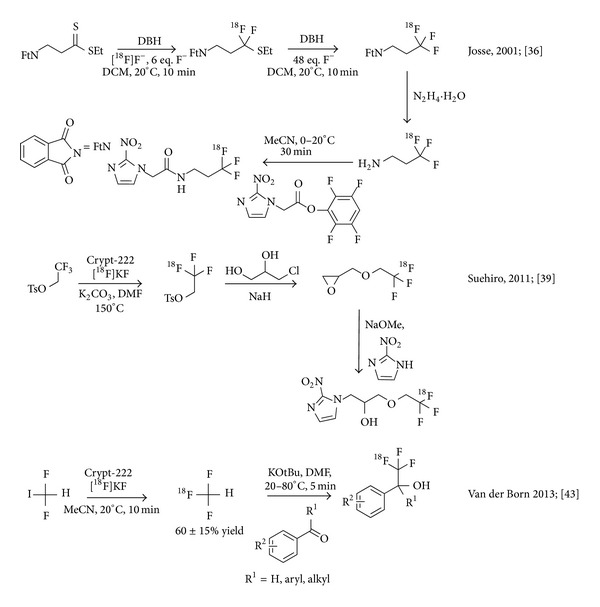
Carrier-added radiosynthesis of ^18^F-labelled hypoxia imaging agents using ^18^F-fluoro-desulfurisation and ^18^F-for-^19^F isotopic exchange and no-carrier-added nucleophilic radiosynthesis of [^18^F]CHF_3_.

A new approach to obtain the title motif of ^18^F-labelled trifluorinated carbon centres was introduced by Josse et al. in 2001 and utilized in the synthesis of ^18^F-labelled 2-(2-nitroimidazol-1-yl)-*N*-(3,3,3-trifluoropropyl)-acetamide ([^18^F]EF3), a prospective PET radiotracer for tissue hypoxia. [^18^F]EF3 was prepared in 3 steps from [^18^F]fluoride ion via 3,3,3-[^18^F]trifluoropropylamine. This ^18^F-labelled building block was obtained in 40% overall chemical yield by oxidative ^18^F-fluorodesulfurization of ethyl N-phthalimido-3-aminopropane dithioate, subsequent deprotection of the intermediate followed by coupling with 2,3,5,6-tetrafluorophenyl 2-(2-nitroimidazol-1-yl) acetate in 5% radiochemical yield within 90 min from cyclotron produced [^18^F]fluoride ion [19]. Specific activity of the final product was limited due to the involvement of a stoichiometric fluoride source in the original desulfurization protocol. Later on the protocol was extended to accommodate a wider spectrum of substrates, namely, triethyl orthothioesters and dithioorthoesters (Figure 3) [45].

2-nitroimidazoles, such as [^18^F]EF3, are used to detect hypoxia, based on the bioreductive metabolism of the nitroimidazole pharmacophore. This metabolism pathway leads to the formation of covalent bioconjugates between intracellular proteins and the imidazole core, which trap the radiotracer within the metabolizing cells. Even though the target compound was obtained in fairly low specific activity, this is not a concern in the study of hypoxia. Indeed, hypoxic tissues can accumulate considerable amounts of substance of nitroimidazoles via an oxygen-level dependent reductive mechanism [46].

Trifluoromisonidazole (1-(2-nitro-1*H*-imidazol-1-yl)-3-(2,2,2-trifluoroethoxy) propan-2-ol, TFMISO) has been considered as a nuclear magnetic resonance imaging (MRI) agent to visualize hypoxic tissues. TFMISO was successfully labelled with ^18^F in an attempt to obtain a bimodal PET/MRI probe. In this report, ^18^F-labeling was achieved via 2,2,2-[^18^F]trifluoroethyl* p*-toluenesulfonate prepared by ^18^F-^19^F exchange. This reagent was used for* O*-^18^F-trifluoroethylation of 3-chloropropane-1,2-diol sodium salt to obtain 1,2-epoxy-3-(2,2,2-[^18^F]trifluoroethoxy)propane. [^18^F]TFMISO was obtained in approximately 40% conversion by ring-opening of the epoxide with 2-nitroimidazole. The researchers “identified 2,2,2-[^18^F]trifluoroethyl tosylate as an excellent [^18^F]trifluoroethylating agent, which can convert efficiently an alcohol into the corresponding [^18^F]trifluoroethyl ether” (Figure 3) [21]. Evidently, the isotopic exchange mechanism limited specific activity in congruence with the amount of added carrier.

The utility of an ^18^F-labelled reagent that could be used as an [^18^F]trifluoromethyl source for a wide variety of different reactions was recognised by Herscheid et al. who reported several nucleophilic approaches to obtain [^18^F]trifluoromethyl iodide and [^18^F]trifluoromethane, respectively, at the biannual symposium of the International Society of Radiopharmaceutical Sciences [47, 48]. This research ultimately resulted in a new strategy towards [^18^F]trifluoromethyl-containing compounds via [^18^F]trifluoromethane developed by van der Born et al. Gaseous [^18^F]trifluoromethane was synthesised in solution at room temperature and trapped in a second reaction vessel. Two further applications of the reagent have been described; [^18^F]trifluoromethane was subsequently used in a reaction with carbonyl compounds to obtain [^18^F]trifluoromethyl carbinols in good yields. Unfortunately, the authors did not report a specific activity in their communication. The reagent was furthermore successfully employed in a preliminary study of Cu-mediated ^18^F-trifluoromethylation; however, no details are available other than a conference abstract (Figure 3) [23, 49].

**Figure 4:**
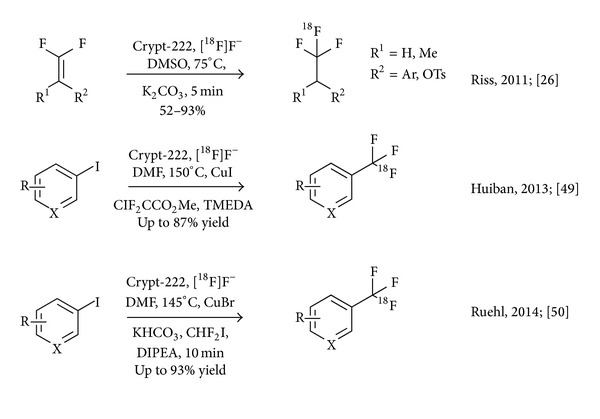
Recent reports on direct nucleophilic radiosynthesis of [^18^F]trifluoroethyl and [^18^F]trifluoromethyl groups.

We devised a procedure for the radiosynthesis of aliphatic [^18^F]trifluoromethyl groups involving the reaction of 1,1-difluorovinyl precursors with [^18^F]fluoride ion, which results in the equivalent of direct nucleophilic addition of H[^18^F]F. In theory, this protocol could make a large pool of trifluoroethylated compounds accessible for the straightforward development of PET radiotracers [22, 50, 51]. The protocol was optimised and applied to a set of substrates with moderate to good outcomes, showing that the method is widely applicable for the synthesis of novel radiotracers. Most notably, 2,2,2-[^18^F]trifluoroethyl* p*-toluenesulfonate was obtained in high radiochemical yields of up to 93% and good specific activity of 86 MBq/nmol starting from a 5 GBq batch of ^18^F. Nevertheless, the reaction requires meticulous control of the reaction conditions and the influence of competing elimination reactions has to be mitigated (Figure 4) [50].

Following the report of an indirect ^18^F-trifluoromethylation reaction by Herscheid and coworkers, Huiban et al. extended a published protocol for direct trifluoromethylation by Su et al. and MacNeil and Burton to ^18^F-radiochemistry [52, 53]. The reaction mechanism involves the formation of difluorocarbene from methyl chlorodifluoroacetate in the presence of a fluoride ion source and CuI. Under no-carrier-added conditions, trace amounts of [^18^F]fluoride ion may only react with a small fraction of the intermediate carbene and form the trifluoromethylating reagent Cu-[^18^F]CF_3_, whereas the bulk of the carbene intermediate degrades to side products and ^19^F fluoride ion. In consequence, the specific activity of the formed product is relatively low (0.1 MBq/nmol). Nevertheless, the method was found to tolerate a variety of substrates and may hence be of use for the study of nonsaturable biological systems (Figure 4) [24].

Seeking an efficient method for producing [^18^F]trifluoromethyl arenes starting from [^18^F]fluoride ion within our radiotracer development program, we have explored a route inspired by the use of [^18^F]fluoroform as an intermediate by van der Born et al. We surmised that reactions involving [^18^F]fluoroform require diligent control of the gaseous intermediate, including low temperature distillation and trapping of the product at −80°C in a secondary reaction vessel. These conditions and technical requirements are limiting factors with respect to the automated synthesis of high activity batches using automated synthesiser systems. In our eyes, widespread adoption of ^18^F-trifluoromethylation reactions would strongly benefit from a straightforward nucleophilic one-pot method generally applicable to the latest generation of synthetic hardware. Such methodology would furthermore feature direct installation of nucleophilic fluorine-18 in the form of no-carrier-added [^18^F]fluoride ion into candidate radiotracers to avoid losses of radioactivity, conserve specific radioactivity, and achieve rapid and simple radiosynthesis. Unfortunately, we were only partially successful; although we have shown that Cu(I) mediated ^18^F-trifluoromethylation reactions are highly efficient in the presence of a simple combination of difluoroiodomethane, DIPEA, CuBr, ^18^F^−^, and an iodo arene, we failed to overcome the known specific activity limitations. Nevertheless, the resulting [^18^F]trifluoromethyl arenes are obtained in sufficient yields of up to 93% in an operationally convenient protocol, suitable for straightforward automation (Figure 4) [25].

**Figure 5:**
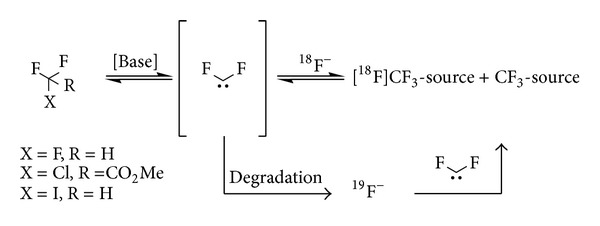
Base mediated *α*-elimination to yield difluoromethyl carbene and subsequent conversion into an ^18^F-trifluoromethylating reagent.

From a mechanistic point of view, the feasibility of the radiosynthesis of [^18^F]trifluoromethylating agents such as [^18^F]fluoroform or Cu-[^18^F]CF_3_ in high specific activity is questionable. In essence, the generation of the aforementioned labelling reagents would require a clean nucleophilic substitution of an appropriate leaving group, which is not likely to occur. Instead, the generic reaction mechanism involves an *α*-elimination in the presence of base to yield difluoromethyl carbene, which is subsequently scavenged by [^18^F]fluoride ion in solution. The inherent low concentration of [^18^F]fluoride ion, combined with the short half-life of difluorocarbene, which degrades to side products under liberation of two equivalents of ^19^F in this pathway directly results in isotopic dilution, which confounds achieving a high specific activity. (Figure 5) [24, 25, 52–54].

Likewise, the intermediate organometallic reagents such as Cu-CF_3_ are unstable in solution; again degradation occurs via a carbenoid pathway. In consequence, several research groups have resorted to the deliberate addition of a soluble source of fluoride ion under stoichiometric conditions to stabilise the equilibrium between the carbene intermediate and the desired reagent, thus adding further indirect proof of the mechanistic difficulties [34, 35].

## 3. Electrophilic Radiosynthesis

Electrophilic methodology has been employed in the synthesis of ^18^F-labelled perfluoroalkyl moieties, for example, in the radiosynthesis and evaluation of the hypoxia imaging agent 2-(2-nitro-1[*H*]-imidazol-1-yl)-*N*-(2,2,3,3,3-[^18^F]pentafluoropropyl)-acetamide ([^18^F]EF-5) [26]. EF-5 is a compound belonging to the class of 2-nitroimidazoles. In this case, the requirements for specific activity are fairly forgiving and radiolabelling using electrophilic fluorine sources with low specific activity may be considered as a viable alternative to [^18^F]fluoride ion. Consequently, stoichiometric reaction conditions are employed and the starting materials are consumed entirely over the course of the labelling reaction. Addition of [^18^F]F_2_ to perfluoroolefins appears to be the method of choice in this context and yields up to 17% of [^18^F]EF-5 have been achieved using carrier-added, gaseous ^18^F-fluorine (Figure 5). Subsequently, Kachur et al. described an improvement of the electrophilic addition of fluorine to a fluorinated double bond, which was achieved by the addition of catalytic amounts (0.5–1%) of boron trifluoride, bromine, or iodine, to the reaction mixture. Under these conditions the conversion of the labelling precursor to [^18^F]EF-5 was improved by 50% [55].

More recently, a simplified procedure for radiosynthesis of [^18^F]EF5 in trifluoroacetic acid (TFA) was devised. This new protocol allowed for straightforward automation of the production using a commercially available radiosynthesis module for routine synthesis of [^18^F]EF-5 in sufficient amounts and purity for clinical PET studies. An evaluation of the radiotracer was conducted in HCT116 xenografts with small animal PET [56].

**Figure 6:**
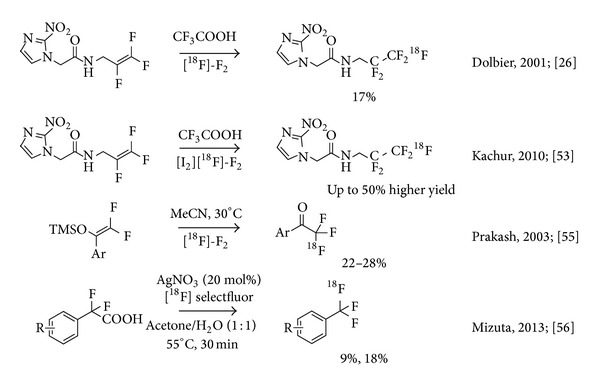
Electrophilic approaches for the radiosynthesis of the title function.

Another example of the use of low specific activity ^18^F-F_2_ under stoichiometric conditions for the synthesis of the CF_3_-motif is the syntheses of several ^18^F-labelled *α*-trifluoromethyl ketones reported by Prakash et al. [27]. Reactions of 2,2-difluoro-1-aryl-1-trimethylsiloxyethenes with [^18^F]F_2_ at low temperature were reported to afford ^18^F-labelled *α*-trifluoromethyl ketones in moderate to good yields within 35–40 min from the end of bombardment. Decay-corrected, isolated yields (>99% radiochemical purity) were reported to fall between 22–28% for radiolabeled model compounds. Specific activities ranged from 0.015–0.020 MBq/nmol at the end of synthesis. This method may be of use for the radiochemical synthesis of biologically active ^18^F-labelled *α*-trifluoromethyl ketones, however, with strict limitations since such low specific activities may confound tracer conditions for PET imaging of saturable biological processes (Figure 6).

Nevertheless, electrophilic fluorination may be the key approach to overcome the issues observed with nucleophilic fluorination attempts. This is owed to the availability of somewhat higher specific activity electrophilic labelling reagents derived from [^18^F]fluoride ion in selected PET centres. A recent report on the reaction of readily available *α*,*α*-difluoro- and *α*-fluoroarylacetic acids with [^18^F]selectfluor bis(triflate) makes accessible the corresponding [^18^F]tri- and [^18^F]difluoromethylarenes in two orders of magnitude higher specific activity compared to [^18^F]F_2_.

This straightforward silver(I) catalyzed decarboxylative fluorination reaction afforded a broad range of [^18^F]trifluoromethyl arenes in moderate to good yields. These researchers reported a specific activity of about 3 MBq/nmol which is about twofold higher than the reported values for nucleophilic fluorination reactions (Figure 6) [28].

## 4. Conclusion

In conclusion, the trifluoromethyl motif has attracted a veritable interest from PET chemists as a relatively abundant fluorinated functional group, which has precipitated in a variety of novel labelling methods, some of which have been used to synthesise radiotracers for PET imaging studies. The major shortcoming of the available methodology is the low specific activity, which impedes PET imaging of saturable processes and may confound widespread application of these methods. Hence, continued effort may be warranted to overcome the limited scope of the available protocols and extend the knowledge base in PET chemistry with new methodology suitable for the radiosynthesis of ^18^F-labelled trifluoromethyl groups in high specific activity.

## Figures and Tables

**Table 1:** Survey of radiosynthetic approaches towards the radiosynthesis of [^18^F]trifluoroalkyl groups.

Method	Year	Yield/%	As (reported value) comment	Reference
(1) ^18^F-^19^F exchange	1979	0.5–15	Low—carrier added	Ido et al. [10]
(2) Sb_2_O_3 _catalysed ^18^F-Cl substitution	1986	20–50	Low—carrier added	Angelini et al. [11, 12]
(3) ^18^F-^19^F exchange	1990	85	Low—(0.00002–0.002 MBq/nmol) carrier added	Kilbourn and Subramanian [13]
(4) ^18^F-^19^F exchange	1993	78	Low—carrier added	Aigbirhio et al. [14]
(5) ^18^F-^19^F exchange	1994	15–99	Low—(0.2–16.6 MBq/nmol) [15] carrier added	Satter et al. [16]
(6) ^18^F-Br substitution	1990	17–28	Low—(0.037 MBq/nmol) precursor separation	Kilbourn et al. [17]
(7) ^18^F-Br substitution	1993	1–4	Low—(1.5–2.5 MBq/nmol) side reaction	Das and Mukherjee [15]
(8) ^18^F-Br substitution	1995	45–60	Low—(0.040–0.800 MBq/nmol) side reactions	Johnstrom and Stone-Elander [18]
(9) ^18^F-fluorodesulfurisation	2001	40	Low—(0.000002 MBq/nmol) carrier added	Josse et al. [19]
(10) ^18^F-Br substitution	2007	10 ± 2	Low—(4.4 ± 1.5 MBq/nmol) side reactions	Prabhakaran et al. [20]
(11) ^18^F-^19^F exchange	2011	~60	Low—carrier added	Suehiro et al. [21]
(12) H^18^F addition	2011	52–93	Moderate (86 MBq/nmol)—side reaction	Riss and Aigbirhio [22]
(13) ^18^F-I substitution	2013	60 ± 15	Not given	Van der Born et al. [23]
(14) Nucleophilic trapping of difluorocarbene formed in situ and Cu(I) mediated trifluoromethylation with Cu-[^18^F]CF_3_	2013	5–87	Low—(0.1 MBq/nmol) side reactions	Huiban et al. [24]
(15) ^18^F-I substitution, in situ formation of Cu-[^18^F]CF_3_	2014	12–93	Low—(0.15 MBq/nmol) side reactions	Ruehl et al. [25]
(16) ^18^F-F_2_ addition	2001	10–17	Low—carrier added	Dolbier et al. [26]
(17) ^18^F-F_2_ disproportionation	2003	22–28	Low—(0.015–0.020 MBq/nmol) carrier-added	Prakash et al. [27]
(18) ^18^F-selectfluor bis-triflate	2013	9–18	Low—(3.3 MBq/nmol) carrier-added	Mizuta et al. [28]
